# Generating Medical Assessments Using a Neural Network Model: Algorithm Development and Validation

**DOI:** 10.2196/14971

**Published:** 2020-01-15

**Authors:** Baotian Hu, Adarsha Bajracharya, Hong Yu

**Affiliations:** 1 Department of Computer Science University of Massachusetts Lowell Lowell, MA United States; 2 Department of Medicine University of Massachusetts Medical School Worcester, MA United States; 3 Bedford Veterans Affairs Medical Center Bedford, MA United States; 4 School of Computer Science University of Massachusetts Amherst Amherst, MA United States

**Keywords:** electronic health record note, medical assessment generation, deep neural network model, artificial intelligence, natural language processing

## Abstract

**Background:**

Since its inception, artificial intelligence has aimed to use computers to help make clinical diagnoses. Evidence-based medical reasoning is important for patient care. Inferring clinical diagnoses is a crucial step during the patient encounter. Previous works mainly used expert systems or machine learning–based methods to predict the International Classification of Diseases - Clinical Modification codes based on electronic health records. We report an alternative approach: inference of clinical diagnoses from patients’ reported symptoms and physicians’ clinical observations.

**Objective:**

We aimed to report a natural language processing system for generating medical assessments based on patient information described in the electronic health record (EHR) notes.

**Methods:**

We processed EHR notes into the Subjective, Objective, Assessment, and Plan sections. We trained a neural network model for medical assessment generation (N2MAG). Our N2MAG is an innovative deep neural model that uses the Subjective and Objective sections of an EHR note to automatically generate an “expert-like” assessment of the patient. N2MAG can be trained in an end-to-end fashion and does not require feature engineering and external knowledge resources.

**Results:**

We evaluated N2MAG and the baseline models both quantitatively and qualitatively. Evaluated by both the Recall-Oriented Understudy for Gisting Evaluation metrics and domain experts, our results show that N2MAG outperformed the existing state-of-the-art baseline models.

**Conclusions:**

N2MAG could generate a medical assessment from the Subject and Objective section descriptions in EHR notes. Future work will assess its potential for providing clinical decision support.

## Introduction

Electronic health record (EHR) systems have been widely adopted by hospitals in the United States and other countries [1], resulting in an unprecedented amount of digital data or EHRs associated with patient encounters [2]. The primary function of EHRs is to document patients’ clinical information and share them among health care providers for patient care. Rich clinical information is represented in the EHRs. In recent years, secondary use of EHRs has helped advance EHR-related computational approaches [3,4].

EHR notes are written by providers who care for their patients. Providers are trained to write notes with a problem-oriented SOAP (Subjective, Objective, Assessment, and Plan) structure [5] along with the Header, which records patients’ necessary information such as name, date of birth, and reason for visit or chief complaint. Textbox 1 shows an illustrative example of a SOAP note for an outpatient encounter. Typically, the subjective section describes patients’ current condition(s), either as patients’ self-reports or physicians’ summaries of previous and pertinent clinical conditions relevant to the chief complaints. This includes medical history, surgical history, family history, and social history along with current medications, smoking status, and drug/alcohol/caffeine use. The Objective section includes clinical conditions, measurements, and observations from patients’ laboratory, physical, and other examinations that are noted during the clinic visit when the note was created. The assessment section typically contains medical diagnoses and summaries of the key elements that lead to the medical diagnoses. Following the diagnoses, physicians lay out the plan for treatment or differential diagnosis, including ordering labs (for differential diagnosis), radiological referrals, performing procedures, and prescribing medications.

**A typical SOAP (Subjective, Objective, Assessment, and Plan) electronic health record note (deidentified).**

**Header:** Umass memorial medical center patient:<patient name> <acct.#> <mr#> <date of birth> <date of service> <address> <physician name> <dictation date> clinic note reason for visit: postoperative visit status post open reduction and percutaneous pinning of right small finger metacarpal neck fracture.

**Subjective:** this is a very pleasant 28-year-old gentleman that we have been following and treating for right small finger metacarpal neck fracture sustained on 03/04/2016 . he feels well . he has been working very closely with hand therapy . he has increased his extension of his small finger. he has not really worked on his grip as of yet .

**Objective:** physical examination: the scar is well healed externally , although it does feel like there is some prominent scar tissue in the deep soft tissues . he is able to better extend his small finger , although there is still a small amount of extensor lag at rest. his sensation otherwise is intact on the radial and ulnar aspects of his finger . radiographs : three views of his hand are taken today and his metacarpal appears better aligned compared to before . he has exhibited bony healing and on the whole , the alignment is acceptable .

**Assessment:** healing well status post open reduction and percutaneous pinning of right small finger metacarpal fracture.

**Plan:** the patient should continue working with hand therapy and at this point, he is 8 weeks out. he may begin some light strengthening with a target date for weightbearing around the 10 to 12-week mark. I have advised him that if it bothers him that he cannot fully extend his small finger secondary to scar tissue, we can always try to perform a tenolysis of the tendon in the future. He wishes to hold off on this and I will plan to see him back in about 2 moths.

Rich clinical knowledge can be inferred from EHRs with such a SOAP structure. In this case, the chief complaint and subjective evidence lead to objective measurements. Assessments are inferred from both subjective and objective evidence and lead to specific plans. As illustrated in Textbox 1, the assessment typically contains two components: (1) a summary of the main conditions, and (2) the diagnoses or likely diagnoses, typically in order from the most likely to the least likely.

Inferring clinical diagnoses is a crucial step during the patient encounter. In the clinical domain, natural language processing (NLP) apps have mainly focused on adverse event detection [6], named entity recognition [7], and relation identification [8]. A closely related system is automated International Classification of Diseases (ICD) code assignment, where these models employ machine learning approaches to predict ICD-Clinical Modification (CM) codes [9]. However, ICD-CM codes are created mainly for billing purposes and have limitations (eg, incomplete assignment [10]) when used as the gold standard for diagnosis labels. In this study, we propose a complementary approach. We built an expert system by directly learning clinical knowledge from SOAP notes to generate medical assessments and diagnoses. Unlike previous expert systems that mainly comprise predefined diagnosis categories, our system generates assessment that is described in natural language.

Automatically generating medical assessment is a challenging task in both computer science and medicine. Both subjective and objective components in a SOAP note are generally verbose, containing abundant medical jargon, much of which is sparse (with low term frequency) and therefore considered as out-of-vocabulary words. EHR narratives also use irregular natural language, including broken sentence structures, and are written by different physicians with different writing styles, many of whom have been trained outside the United States.

Our computation model for medical assessment generation is based on our observation that the medical assessment generation task is partially analogous to the abstractive text summarization tasks. In recent years, much progress has been made on neural abstractive summarizations [11]. The canonical neural sequence-to-sequence model uses recurrent neural network (RNN) to encode an input document and another RNN as a decoder with an attention mechanism to generate the target text [12]. State-of-the-art models have been proposed in recent years, such as the copy mechanism [13,14] and coverage mechanism [15]. These models have demonstrated advances for generating long-document summarization [16].

In this study, we explored these aforementioned state-of-the-art models as baseline models for Assessment generation. Our innovative approach is as follows: In addition to depending on the Subjective and Objective descriptions, the Assessment generation is conditioned on the chief complaint(s), which is the reason that a patient seeks medical treatment. Therefore, our NN model for medical assessment generation (N2MAG) augments the pointer-generator network proposed by Seeet al [16], with an innovative attention-over-attention model. Thus, the chief complaints information in the Header section could be used to infer assessment. Evaluation of 953 patients’ EHR notes shows that N2MAG can generate natural and fluent assessment, significantly outperforming competitive baseline models by using both the Recall-Oriented Understudy for Gisting Evaluation (ROUGE) evaluation metrics and physicians’ evaluation.

## Methods

### The Overall Architecture

**Figure 1:**
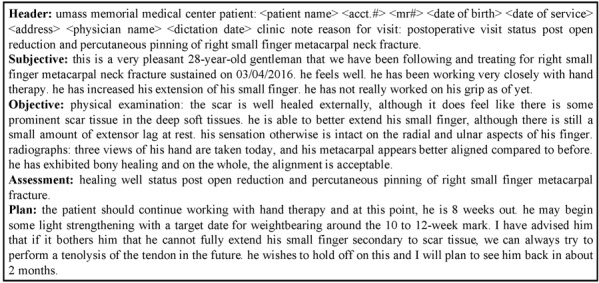
Illustration of the Neural Model for Medical Assessment Generation (N2MAG).

N2MAG merges the narrative text *X* in subjective and objective sections as an input document, denoted as a sequence of words (f_1_, f_2_...f_n_). Its header section, *T,* is represented by a sequence of words (w_1_, w_2_...w_m_). The goal of N2MAG is to generate the assessment, *Y*, consisting of a word sequence (y_1_, y_2_...y_l_), given *X* and *T*. As illustrated in Figure 1, N2MAG has three components: the encoder of subjective and objective sections (the main encoder), the encoder of the header section, and the decoder that generates medical assessment.

This study obtained approval from the Institutional Review Board at the University of Massachusetts Medical School.

### The Main Encoder

The N2MAG uses a single-layer, bidirectional long short-term memory (LSTM) neural network [17] to encode the input text (ie, the subjective and objective sections). LSTM is commonly used for sequence-related applications [11,18]. The sequence of words in subjective and objective sections *X* is first mapped to a sequence of word vectors (x_1_...x_n_), by looking up the word embedding matrix M^dx|V|^, where d denotes the dimension of word embeddings and |V| denotes the size of vocabulary. The word vector *x_i_* is then fed into the bidirectional LSTM (denoted as LSTM_source_) one by one, which produces a sequence of encoder hidden states [h_1_…h_n_], denoted as H. The subjective and objective text is therefore represented as a sequence of hidden states *H*.

### The Encoder of the Header Section

For the canonical neural sequence to sequence model, there is only one encoder, that is, LSTM_source_. However, for medical assessment generation, the Header section contains valuable information (eg, chief complaints), which is useful for assessment generation. In order to encode the Header section, N2MAG uses another bidirectional LSTM denoted as LSTM_header_. Similar to the encoder of the subjective and objective sections, the sequence of words in the Header section *T* is first mapped to a sequence of word vectors (t_1_…t_m_) denoted as *T*. The word vector *t_i_* is then fed into the encoder LSTM_header_ one by one, which produces a sequence of encoder hidden states [z_1_…z_m_], denoted as *Z*:

Z=LSTM_header_ (t_1_...t_m_) (1)

For N2MAG, *Z* will be used by the decoder to fetch more accurate information from the subjective and objective input sections.

### The Decoder of Assessment

The decoder of N2MAG is a single-layer LSTM. It generates words one by one from the given start symbol </begin> and terminates when </end> is generated or the maximum decoding length is reached. At each step, the decoder LSTM receives the word embedding of the previous word to produce the decode state s_i_.

The decoder of N2MAG first uses s_i_ to attend to the hidden states *Z* of the Header section encoder. The attention distribution on *Z* can be calculated as Equation 2, where *z_j_* is the encoder hidden state of the jth word in the header section.


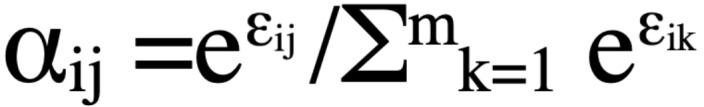
 (2)

ε_ij_=V^T^tanh(W_Z_z_j_+W_S_s_j_+b_z_) (3)

The patient’s information z_i_^*^, which the decoder attended to during the decoding step *i*, can be calculated as Equation 4:

z_i_^*^=Σ^m^_k=1_α_ik_ z_k_ (4)

where V, W_Z_, W_S_, and b_Z_ are learnable parameters.

In the next step, N2MAG uses *s_i_* and z_i_^*^ to attend to the hidden states *H*. The attention probability of *h_j_* on the decoding step *i* is calculated as Equation 5. The attention distribution β_i*_ of *H* on the decoding step *i* can be represented as (β_i1_...β_in_).


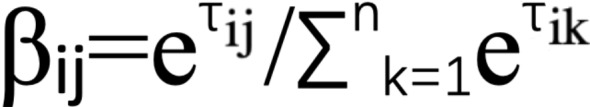
 (5)


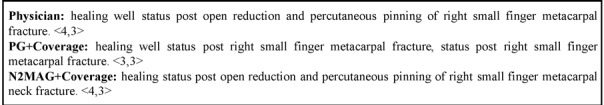
 (6)

where 
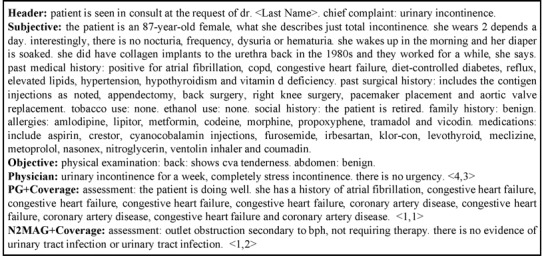
 are learnable parameters.

N2MAG uses the attention distribution β_i*_ to fetch information h_i_^*^ from the subjective and objective sections, which can be calculated as mentioned in Equation 7:

h_i_^*^=Σ^n^_k=1_β_ik_h_k_ (7)

This equation allows N2MAG to consider both the current decoder state and the patient’s information to fetch information from the subjective and objective sections, which can be viewed as the attention-over-attention mechanism. Generally, the current decoder state s_i_ is to inform the decoder of which types of information are to be fetched. The z_i_^*^ forces the decoder to target at a more specific location.

To handle out-of-vocabulary words in EHR notes, N2MAG also uses copying or pointing mechanisms [13,14]. The copying mechanism allows the network to copy words from the source text. N2MAG first computes the probability *p^i^_gen_* of generating a word from the predefined vocabulary on decoding step *i*, which can be formulated as Equation 8.

p^i^_gen_=σ(W’_h*_h^*^_i_+ W’_S_s_i_+ W’_y_ y_i-1_+b’) (8)

where W’_h*_, W’_S_, W’_y_, and scalar b’ are learnable parameters; *p^i^_gen_* is then used as a soft gate to decide whether to sample a word from the distribution on predefined vocabulary or from the attention distribution β_i*_. The final probability of the word *w* output by the decoder on decoding step *i* can be formulated as Equation 9:

p^i^(w)= p^i^_gen_ * p^i^_voc_(w)+(1- p^i^_gen_)*Σ^n^_j=1_1(w_j_=w)* β_ij_ (9)

where 1(w_j_=w) equals to 1, if the *j*th word is in the subjective and objective section *X* and is the word *w*. Otherwise, 1(w_j_=w) equals to 0; *p^i^_voc_(w)* is the probability of sampling word *w* from the predefined vocabulary on decoding step *i*; and *p^i^_voc_* is the word distribution on predefined vocabulary on decoding step *i*, which can be computed in Equation 10:


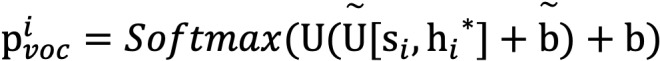
 (10)

where 
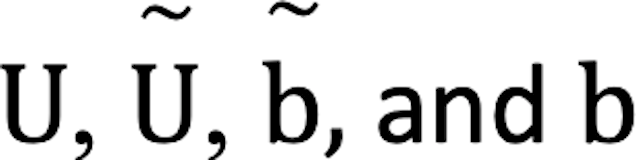
 are learnable parameters.

In summary, our N2MAG uses both the attention-over-attention and copying mechanisms. The attention-over-attention can facilitate the decoder to locate more accurate information from the narrative text. The copying mechanism can alleviate the out-of-vocabulary problems during decoding.

### Training

The parameters *θ* of the N2MAG includes four parts: the word embedding matrix *M*, the parameter *θ_1_* of 
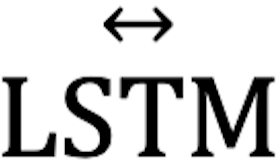
_source_, the parameter *θ_2_* of 
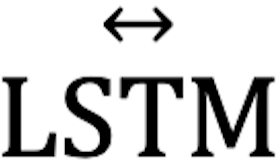
_header_, and the parameter *θ_3_* for the decoder of assessment. The probability of generating reference assessment *Y* can be formulated in Equation 11:

P(Y|X,T; θ)=∏^l^_i=1_P^i^(y_i_) (11)

The negative log-likelihood loss for generating the reference assessment *Y* is calculated as Equation 12:

Loss_nll_(Y|X,T;θ)=–Σ^l^_i=1_log(P^i^(y_i_))/l (12)

Equation 12 is the basic loss used in N2MAG. Our loss function is based on the recent research on the neural sequence-to-sequence models such as minimum risk training [19], cost weighting [20], and coverage mechanism [15]. Since clinical content integrity is very important for making a diagnosis, we chose the coverage mechanism, which forces the model to attend to the different locations of source text instead of one. On the decoding step *i*, the decoder uses the Equation 13 mentioned below to compute the vector (c_i1_…c_in_) denoted as c_i*_, whose dimension equals the length of the subjective and objective text. In addition, c_i*_ is used to record the accumulative attention degree of each word until the decoding step *i*:

c_i*_=Σ_k=1_^i-1^β_i*_ (13)

Then, c_i*_ is added to equation 6 as an extra factor. Hence, equation 6 is modified to Equation 14 as follows:


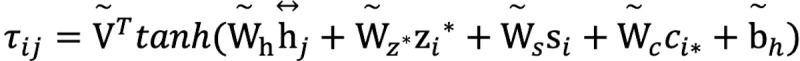
 (14)

where 
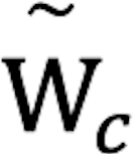
 is the extra learnable parameter. Therefore, in the training period, the learnable parameter θ’ includes two parts 
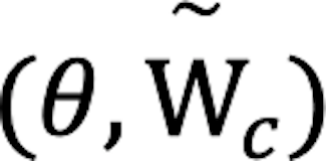
. We use the coverage loss Loss_cov_ as Equation 15:

Loss_cov_(Y|X,T;θ^’^)= Σ^l^_k=1_Σ^n^_j=1_ min(β_kj,_ c_kj_) (15)

Finally, the coverage loss Loss_cov_ and negative log-likelihood loss Loss_nll_(Y|X,T;θ) are linearly combined with hyperparameter λ as Equation 16.

Loss(Y|X,T; θ^’^)= Loss_nll_(Y|X,T;θ)+λLoss_nll_(Y|X,T;θ^’^) (16)

The λLoss_nll_(Y|X,T;θ^’^) can be viewed as the model regularization factor. It can prevent N2MAG from overfitting on specific local parts. In practice, we first train N2MAG with the loss Loss_nll_(Y|X,T;θ) until it converges on the validation set. Subsequently, we incorporate the coverage mechanism into pretrained N2MAG and continue to train it with the loss Loss(Y|X,T;θ^’^).

### Experiments and Systems

#### Dataset

Our EHR data comprise 235,458 outpatient EHR notes from the University of Massachusetts Memorial Medical Center, from which we randomly selected 233,470, 1,035, and 953 notes for training, development, and test sets, respectively. As described previously, a typical structure of EHR notes includes the Header and SOAP sections, as shown in Textbox 1, although variations exist. For example, in some notes, Subjective and Objective sections are not explicitly marked, but the relevant content is described in other sections such as “History of present illness.” To address the variations, we simply aggregated the text between “History of present illness” and “Assessment” as the “Subjective” and “Objective” sections.

#### Models

We compare N2MAG with the state-of-the-art neural sequence-to-sequence models. The detailed setups of the baseline and our N2MAG models are described as follows:

- Seq2Seq+att: Seq2Seq+att is the model proposed by Bahdanau et al [12], which is commonly used as the benchmark model for sequence-to-sequence tasks.
- Pointer-generator (PG): PG [16] is the state-of-the-art model for document summarization. It incorporates the copying mechanism on the Seq2Seq+att model.
- PG+Coverage: PG+Coverage is proposed by See et al [16]. It incorporates the coverage mechanism based on the pretrained PG. The hyperparameter λ is set to 0.2.
- N2MAG: N2MAG is trained with negative likelihood loss Lossnll(Y|X,T;θ).
- N2MAG+Coverage: It incorporates the coverage mechanism based on the pretrained N2MAG and is continuously trained with loss Loss(Y|X,T; θ’). The hyperparameter λ is set to 0.2.

#### Settings

All aforementioned models use LSTM as both the encoder and decoder to train on the same training set. All the hyperparameters are chosen empirically. The dimension of the hidden state is set to 200, and the embedding dimension is set to 128. All the parameters are randomly initialized. The vocabulary size is set to 100,000. We take the tokens that contain digit as out-of-vocabulary words and add the digit “0-9” to the vocabulary. During training and testing, we truncate the subjective and objective sections to 500 tokens and limit the length of the assessment section to 60 tokens for training. For N2MAG and N2MAG+Coverage, we truncate the Header section to 100 tokens. All these models are trained using Adagrad [21] with a learning rate of 0.12 and an initial accumulator value of 0.11. We use the loss on the validation set to implement early stopping [22]. At the test time, all the models produce assessment using beam search with a beam size of 10, the minimum decoding length is set to 15, and the maximum decoding length is set to 60.

### Evaluation

#### Recall-Oriented Understudy for Gisting Evaluation

Recall-Oriented Understudy for Gisting Evaluation (ROUGE) [23] is commonly used to evaluate document summarization models and has been proven to be strongly correlated with human evaluation results. We therefore use ROUGE to evaluate N2MAG and other baseline models.

There are multiple variants of ROUGE scores. Among them, ROUGE-1 (R-1), ROUGE-2 (R-2), and ROUGE-L (R-L) are the most commonly used ones. ROUGE-n (R-n) can be computed as Equation 17 below:


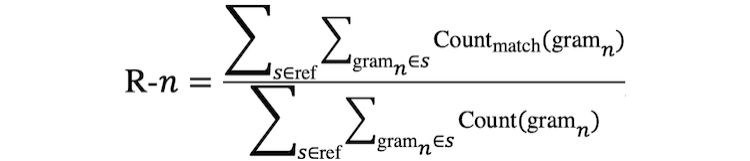
 (17)

where *n* stands for the length of the n-gram, Count_match_(gram_n_) is the maximum number of n-grams co-occurring in both the generated assessment and the reference. Similarly, we could compute the R-n precision and F_1_. R-1 and R-2 are special cases of R-n, in which n=1 or n=2. R-L is instead computed based on the length of the longest common subsequence between the candidate assessment and the reference. In this work, we use F_1_ of R-1, R-2, and R-L as our evaluation.

#### Expert Evaluation

We also conducted a qualitative evaluation to compare the N2MAG+Coverage model with the PG+Coverage model, since both models have competitive performance based on our quantitative evaluation results. We randomly sampled 50 patients’ EHR notes from the test set and asked two unbiased physicians who were not privy to the reasons, to evaluate the quality of the generated assessments. Specifically, for each EHR note, we presented three assessments (the doctor’s assessment, assessments produced by N2MAG+Coverage, and PG+Coverage) to two physicians. To ensure fairness, the order of the three assessments for each EHR note was randomized. In order to eliminate bias against computer-generated outputs, we informed the physician evaluators that all three assessments are outputs by a machine. The score ranged from 1 to 5, where 1 denotes “the worst” and 5 denotes “the best.”

## Results

Table 1 shows the performance comparison between our models and the baseline models. The results show that both N2MAG and PG with the copying mechanism outperformed the Seq2Seq+att model. Our manual analysis concluded that the copying mechanism can mitigate data sparsity. Specifically, even with a large vocabulary, the Seq2Seq+att models failed to generate some words (such as the patient’s name and age), while the models (PG and N2MAG) with copying mechanism could generate these words. Although it is common for doctors to describe patients’ basic information (such as name and age), such information represents the rare word challenge. This is also one of the reasons that Seq2Seq+att performed poorly based on ROUGE.

The results also show that PG+Coverage and N2MAG+Coverage outperformed their corresponding PG and N2MAG models. The results demonstrate that the coverage mechanism can boost the model to comprehend patients’ EHR notes as a whole instead of only focusing on some specific text. These results conclude that both the copying and coverage mechanisms benefit PG and N2MAG performance, which is in line with the previous research in the NLP domain, such as document summarization [13,16] and machine translation [15].

Table 1 shows that both N2MAG and N2MAG+Coverage, which use the attention-over-attention mechanism to incorporate the patients’ basic information, outperformed PG and PG+Coverage. The results support our intuition that patients’ chief complaint information is valuable. For example, in Textbox 1, the “reason for visit” clearly shows that the main purpose of the patient’s visit is “postoperative visit status post open reduction and percutaneous pinning of right small finger metacarpal neck fracture.” Our attention-over-attention mechanism allowed the models to condition on the chief complaint and therefore generated better assessments.

**Table 1:** Performance results evaluated with the F1 ROUGE scores (%). All scores of N2MAG and N2MAG+Coverage are statistically significant using 95% CIs with respect to competitor models.

Model	ROUGE^a^-1	ROUGE-2	ROUGE-L
Seq2Seq+att	37.4	20.3	34.7
PG^b^	38.6	22.5	35.8
PG+Coverage	41.6	24.8	38.6
N2MAG^c^	43.1	27.0	40.2
N2MAG+Coverage	45.2	28.5	41.8

^a^ROUGE: Recall-Oriented Understudy for Gisting Evaluation.

^b^PG: point-generator.

^c^N2MAG: neural network model for medical assessment generation.

Table 2 shows the physician's evaluation results. The results show that N2MAG+Coverage outperformed PG+Coverage based on the overall quality of assessment. The results show that although both PG+Coverage and N2MAG+Coverage achieved better scores on ROUGE, their overall quality scores remained lower (average of 2.17 and 2.36, respectively). On the other hand, the evaluation scores of doctors were also low (average of 2.92). Our results are not surprising, as there is a wealth of literature that has shown low agreement among physicians. In addition, since physician evaluators were informed that all three outputs were generated by computer systems, bias against computer systems may lead to poor overall scores.

**Table 2:** Results of two physicians’ evaluations.

Model	Physician 1	Physician 2	Average
Human	3.14	2.70	2.92
PG^a^+Coverage	2.50	1.84	2.17
N2MAG^b^+Coverage	2.66	2.06	2.36

^a^PG: point-generator.

^b^N2MAG: neural network model for medical assessment generation.

We analyzed the physicians’ evaluation results. We found that for 42 of 50 (84%) assessments, physician evaluators judged that N2MAG+Coverage outperformed PG+Coverage. In addition, for 18 of 50 (36%) assessments, physicians judged that N2MAG+Coverage outperformed or performed equally as the doctor who wrote the assessment of his/her patient.

## Discussion

### Error Analyses

We also conducted error analyses. As described in the Results section, N2MAG+Coverage outperformed PG+Coverage 84% of the time. An example is illustrated in Textbox 2. In this example, all three assessments correctly identified the type of injury, which is a right small finger metacarpal fracture and that the wound was healing. However, only the doctor and N2MAG+Coverage identified the type of surgery the patient underwent, which is open reduction and percutaneous pinning of the fractured bone. The difference is crucial, as the interpretation from human and N2MAG+Coverage assessments would be correct (ie, the patient is recovering after undergoing surgical treatment for the fracture), while the PG+Coverage assessment would be incorrect (ie, the patient is recovering from the fracture [without treatment]). This example shows the importance for attention over attention.

**The generated assessments for the note in Figure 1. The numbers in brackets are the two physicians' scores.**

**Physician:** healing well status post open reduction and percutaneous pinning of right small finger metacarpal fracture. <4,3>

** PG+Coverage:** healing well status post right small finger metacarpal fracture, status post right small finger metacarpal fracture. <3,3>

** N2MAG+Coverage:** healing status post open reduction and percutaneous pinning of right small finger metacarpal fracture. <4,3>

Although the result of ROUGE and expert evaluation demonstrate the utility of our N2MAG models in generating accurate medical assessments, we found that the N2MAG models made a lot of mistakes, many of which were severe, including wrong diagnoses. An example is shown in Textbox 3. The clinical narrative describes a patient’s current problem, which is urinary incontinence. The severity of the problem required the patient to use two diapers a day. The narrative also describes the prior treatment in addition to other medical conditions, surgical treatments, and current medications. Based on clinical knowledge, urinary tract infection can often be present with urinary incontinence. As such, the documented physical examination shows the clinician’s effort to look for findings suggestive of urinary tract infection. Based on the information provided, the patient has urinary incontinence but cannot fully rule out urinary tract infection because the patient has pain in her flank. Upon analysis of the three assessments, only the assessment generated by the doctor identified urinary incontinence. In contrast, PG+Coverage provided no information on the current status of the patient, while N2MAG+Coverage made with a wrong diagnosis of benign prostate hyperplasia, a condition that is not seen in females, and ruled out urinary tract infection. We speculate that if we increase the training size that N2MAG is trained on, we may mitigate this kind of mistake.

**The generated assessments for one electronic health record note. The numbers in brackets are two physicians' scores.**

**Header:** patient is seen in consult at the request of dr. <Last Name >. chief complaint: urinary incontinence.

**Subjective:** the patient is an 87-year-old female, what she describes just total incontinence. she wears 2 depends a day. interestingly, there is no nocturia, frequency, dysuria or hematuria . she wakes up in the morning and her diaper is soaked. she did have collagen implants to the urethra back in the 1980s and they worked for a while, she says. past medical history: positive for atrial fibrillation, copd, congestive heart failure, diet-controlled diabetes, reflux, elevated lipids, hypertension, hypothyroidism and vitamin d deficiency. past surgical history: includes the contigen injections as noted, appendectomy, back surgery, right knee surgery, pacemaker placement and aortic valve replacement. tobacco use: none. ethanol use: none. social history: the patient is retired. family history: benign. allergies: amlodipine, lipitor, metformin, codeine, morphine, propoxyphene, tramadol and vicodin. medications: include aspirin, crestor, cyanocobalamin injections, furosemide, irbesartan, klor-con, levothyroid, meclizine, metoprolol, nasonex, nitroglycerin, ventolin inhaler and coumadin.

**Objective:** physical examination: back: shows cva tenderness. abdomen: benign.

**Physician:** urinary incontinence for a week, completely stress incontinence. there is no urgency. <4,3>

**PG+Coverage:** assessment: the patient is doing well she has a history of atrial fibrillation, congestive heart failure, congestive heart failure, congestive heart failure, congestive heart failure, coronary artery disease, congestive heart failure, coronary artery disease and coronary artery disease. <1,1>

**N2MAG+Coverage:** assessment: outlet obstruction secondary to bph, not requiring therapy, there is no evidence of urinary tract infection or urinary tract infection. <1,2>

Our results show that physician evaluators provided low scores for doctors’ assessments, mainly due to inadequate coverage. For example, in the previous example, our two physician evaluators gave the doctors’ assessment scores of 4 and 3, because both considered that the doctor’s assessment was incomplete: The assessment only described one of the symptoms but failed to describe the possibility of urinary tract infection.

As the world population is living longer, patients are increasingly having more complex diseases. At the same time, physicians are increasingly trained with specializations. We believe that N2MAG may be used as an efficient tool for clinical decision support.

### The Model Interpretation

**Figure 2:**
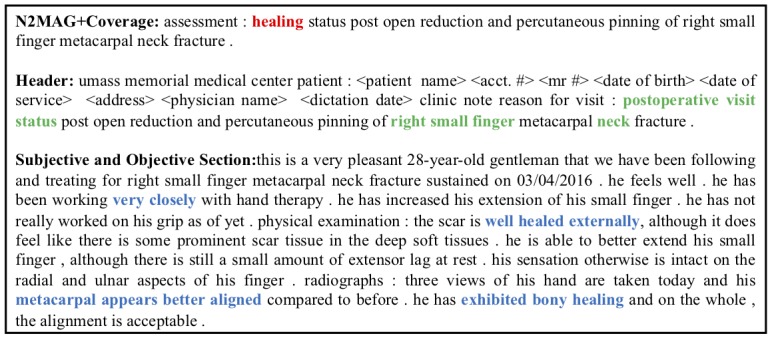
Example for model interpretation.

Interpretability or explainability is crucial for any clinical applications. However, interpretability is typically a well-known challenge for deep neural models. In contrast, our novel attention-over-attention mechanism architecture allows an excellent interpretability. For example, as shown in Figure 2, by analyzing the attention weights for the Header section, when generating the word “healing,” the decoder mainly focuses on the words (green words) “postoperative visit status,” “right small finger,” and “neck” in the Header section. Therefore, these words summarize the main reason why patients visit the physician. Accordingly, the decoder is based on this information and extends to “postoperative visit status,” “right small finger,” and “neck,” from the Subjective and Objective sections. Based on the attention weights for the Subjective and Objective sections, the decoder is shown to mainly pay attention to the words (blue words) “very closely,” “well healed externally,” “metacarpal appears better aligned,” and “has exhibited bony healing.” From these words, we can see that the status of the patient is becoming better. By combining the aforementioned information, the decoder makes a decision to generate and output the word “healing” in the assessment.

### Conclusion and Future Direction

In this paper, we proposed a novel neural model for EHR medical assessment generation (N2MAG). N2MAG takes on input as Subjective and Objective content and conditions of the chief complaint, and outputs Assessment in natural language. Our evaluation results show that N2MAG substantially outperformed other state-of-the-art machine learning models. In addition, a comparison between N2MAG and physician experts has shown that N2MAG performed equally or outperformed doctors in 36% assessments. As the medical domain has become more specialized, N2MAG has the potential to be used to as a clinical decision system by generating a medical assessment draft for physicians. N2MAG could highlight salient information, which may help physicians reduce the information overload burden and improve the efficiency. To improve N2MAG, we will increase the size of EHRs for training to mitigate data sparsity. We will also incorporate external knowledge resources such as clinical guidelines.

## Abbreviations

CNN: convolutional neural network
EHR: electronic health record
LSTM: long short-term memory
N2MAG: the neural network model for medical assessment generation
NLP: natural language processing
R-1: ROUGE-1
R-2: ROUGE-2
R-L: ROUGE-L
RNN: recurrent neural network
ROUGE: Recall-Oriented Understudy for Gisting Evaluation
SOAP: Subjective, Objective, Assessment, and Plan
